# Transcriptome of peanut kernel and shell reveals the mechanism of calcium on peanut pod development

**DOI:** 10.1038/s41598-020-72893-9

**Published:** 2020-09-24

**Authors:** Sha Yang, Jianguo Wang, Zhaohui Tang, Feng Guo, Ye Zhang, Jialei Zhang, Jingjing Meng, Lei Zheng, Shubo Wan, Xinguo Li

**Affiliations:** 1 Biotechnology Research Center, Shandosng Academy of Agricultural Sciences, Jinan, 250100 People’s Republic of China; 2 College of Life and Environment Sciences, HuangShan University, Huangshan City, China; 3 Shandong Academy of Agricultural Sciences/Shandong Provincial Key Laboratory of Crop Genetic Improvement, Ecology and Physiology, Jinan, 250100 People’s Republic of China

**Keywords:** Computational biology and bioinformatics, Genome informatics, Plant sciences, Plant physiology, Plant signalling

## Abstract

Calcium is not only a nutrient necessary for plant growth but also a ubiquitous central element of different signaling pathways. Ca^2+^ deficiency in soil may cause embryo abortion, which can eventually lead to abnormal development of peanut pods during the harvest season. To further study the mechanisms by which Ca^2+^ affects the shells and kernels of peanuts, transcriptome sequencing was used to explore the genes differentially expressed in shells and kernels during the early stage of peanut pod development between Ca^2+^ sufficient and deficient treatments. In this study, 38,894 expressed genes were detected. RNA-seq based gene expression profiling showed a large number of genes at the transcriptional level that changed significantly in shells and kernels between the Ca^2+^ sufficient and deficient treatments, respectively. Genes encoding key proteins involved in Ca^2+^ signal transduction, hormones, development, ion transport, and nutrition absorption changed significantly. Meanwhile, in the early stage of pod development, calcium first promoted nutrient absorption and development of shells, which has less effect on the formation of seed kernels. These results provide useful information for understanding the relationship between Ca^2+^ absorption and pod development.

## Introduction

Peanut (*Arachis hypogaea* L.) is one of the most important widely grown oil-crops, providing 20% of the global cooking oil and 11% of protein production annually^1^. After flowering and fertilization of peanuts, a novel organ called a peg (which is an elongated ovary) forms^2^. The peanut embryo located in the tip of the peg stays in a relatively static state after a few cells differentiate^3^. As the peanut peg elongates, the development of the embryo and pod resumes after the peg is buried into the soil. During this period, the environmental conditions change significantly, and the combination of darkness, mechanical stimuli, moisture, and nutrition together promote the growth and expansion of the pod^4^. The pod buried in the soil can directly absorb moisture and minerals for its growth and development^5^. Previous research has reported that Ca^2+^-deficient soil can induce the abortion of peanut embryos or prevent the expansion of kernel, and this may eventually lead to the reduction of peanut yields. Supplied with sufficient Ca^2+^ would increase the full degree of peanut pod^6^. It has been suggested that Ca^2+^ plays an important role in the development of peanut pods.

Ca^2+^ is an essential plant nutrient that plays a pivotal role in plant growth and development processes, such as cell division, cell polarity, circadian rhythms, stomatal closure, senescence, and responses to multiple stresses^7^. Ca^2+^ deficiency can cause diverse symptoms in horticulture. Tip burn of leafy vegetables during young expanding leaves and brown heart or black heart of leafy vegetables was induced by Ca^2+^ deficiency. Blossom end rot of watermelon, pepper, and tomato fruits and bitter apple pits can be caused by the lack of Ca^2+^ in fertilizers^8^. Owing to the immobility of Ca^2+^ in older tissues, these symptoms tend to occur in developing tissues. Cracking can also occur in tomato, cherry, and apple fruits under a lack of Ca^2+^ and increased humidity and rainfall^9^. These symptoms indicated that Ca^2+^ functions in plant cell walls^10^. Previous studies have shown that Ca^2+^ deficiency can cause empty pods in peanut. Ca^2+^ is transported from roots to transpiration organs mainly through xylem and driven by solar energy^11^. The growth and development of peanut pods occurs underground, where they are unable to absorb Ca^2+^ transported from roots through xylem^12^. Using transcriptomic analysis, several important differentially expressed genes (DEGs) between the aerial pegs and underground swelling pods have been identified in our previous studies^13,14^. We first provided a mechanism by which exogenous Ca^2+^ affects the aerial and underground parts of peanut plants. On the one hand, Ca^2+^ enhanced the storage of aerial nutrients. On the other hand, it activated Ca^2+^ signaling pathways and hormone-related genes in embryonic development^14^. The development processes also differed between the shell and kernel. For example, the dryness of shells was very low when peanuts entered the pod setting stage. With the development of pod, the increasing trend of seed kernel was basically the same as that of shell, but the fast increasing period of seed kernel dryness occurred later than that of shells, with lower total accumulation^15^. Whether the responses of shells and seed kernels to Ca^2+^ deficiency were the same or different requires further study.

Based on the completion of the peanut genome, there has been a new opportunity to understand the genes that are differentially expressed in peanut shell and kernel between Ca^2+^ sufficient and deficient conditions. In this study, using RNA-seq profiling, the differentially expressed genes of peanut shells and kernels were identified under Ca^2+^ sufficient and deficient conditions. This study had three major purposes: (1) to compare the DEGs of shells and seed kernels of peanut plants between free Ca^2+^ sufficient and free Ca^2+^ deficient treatments; (2) to identify candidate genes involved in peanut embryo and shell development processes and affected by Ca^2+^; (3) to clarify the regulatory network involving Ca^2+^ during peanut shell and embryo development processes.

## Results

### Transcriptome sequencing

The purpose of this study was to reveal the molecular mechanisms underlying the effects of free Ca^2+^ on shell and kernel tissues during peanut pod development by using transcriptome sequences. Raw reads ranging from 69.73 to 74.58 M were detected in each sample (Table 1). After removing low quality reads, average clean reads of 68.4 M were acquired in each sample with approximately 6.8 Gb of clean bases obtained. The clean read ratio ranged from 93.46 to 95.37%. Clean reads were used for further analysis. In each sample, a mean of 80.34% of clean reads mapped to the reference genome, and approximately 60% uniquely mapped to the reference genome (Table 1). Random RNA fragmentation would be reflected as an even distribution of read positions across each gene, while the coverage decreased at both the 3′ and 5′ ends (Fig. S1). The percentage of genes with 90–100% coverage was approximately 35%, while more than 20% of genes had 80–90% coverage (Fig. S2). As the sequencing depth increased, the number of detected genes reached saturation (Fig. S3), which suggests that the libraries well represent the transcripts in each sample.

**Table 1:** Summary of read number from pericarps and nutlets align to reference genome.

Sample	Total raw reads (M)	Total clean reads (M)	Total clean bases (Gb)	Clean reads ratio (%)	Total mapping (%)	Uniquely mapping (%)
NS1	74.29	69.88	6.99	94.07	79.97	59.28
NS2	69.73	66.17	6.62	94.91	82.76	61.71
NS3	70.8	67.51	6.75	95.34	82.15	61.14
ND	74.58	71.09	7.11	95.33	80.22	59.3
ND	72.22	68.88	6.89	95.37	82.15	60.66
ND	74.71	70.79	7.08	94.75	82.18	61.05
PS1	72.22	67.95	6.79	94.09	78.35	57.88
PS2	71.55	67.73	6.77	94.65	80.91	59.35
PS3	74.71	69.82	6.98	93.46	81.54	60.31
PD1	69.73	65.5	6.55	93.94	79.37	58.14
PD2	72.22	68.47	6.85	94.81	74.91	54.17
PD3	70.55	66.81	6.68	94.7	79.62	58.32

In this study, total 38,894 genes were detected. Among them, the number of known genes was 35,043 and 3851 genes were predicted as new genes. The number of total new transcripts was 38,891, and among them, 28,817 were new variable splicing subtypes of known protein-coding genes. The remaining 10,074 transcripts were long non-coding RNAs. Five types of variable splicing events were observed, including skipped exon (SE), alternative 5′ splicing site (A5SS), alternative 3′ splicing site (A3SS), mutually exclusive exons (MXE), and retained intron (RI) splice variants, and these results were summarized in Supplemental Fig. S4. To genotype the transcriptome data, GATK software was used to call SNPs and InDels in each sample. Six types of SNP were statistically significant and are listed in Fig. S5. More than 69% of SNP sites occurred in exons, with approximately 13% of SNP sites occurring in introns, and only 2.3% of SNP sites occurred in up and down 2 K (Fig. S6). Resembling the distribution of SNP sites, more than 50% of InDels occurred in exons, with fewer distributed in introns and intergenic regions, and only about 5% of InDels distributed in up and down 2 K (Fig. S7).

### Verifying DEGs in shells and kernels between free Ca^2+^ deficient and sufficient treatments

**Figure 1:**
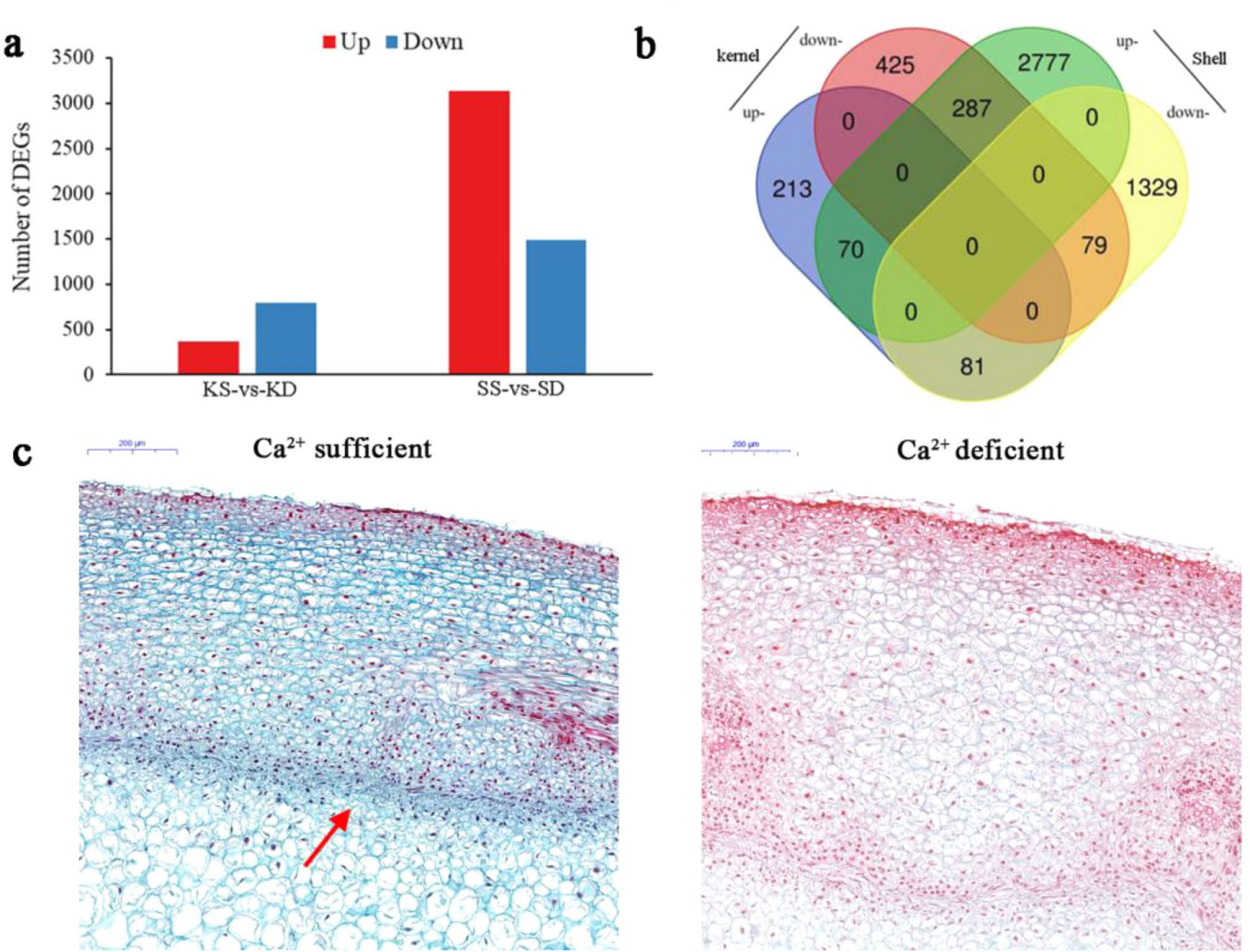
Differently expressed genes in ND, NS, PD and PS. (**a**) Numbers of differently expressed genes in response to free Ca^2+^ treatment. (**b**) Venn diagram demonstrated the common and specific differently expressed genes in kernel and shell. (**c**) Histochemical analysis of peanut shell cells under Ca^2+^ sufficient or deficient treatments. The arrows indicate the areas where the shells differ significantly between Ca^2+^ sufficient or deficient treatments.

To obtain gene expression profiles throughout peanut pod development, twelve cDNA libraries were constructed using kernels and shells under Ca^2+^ sufficient (referred to as KS and SS) and Ca^2+^ deficient treatments (referred to as KD and SD), respectively. A total of 1151 and 6423 DEGs were observed in kernels and shells, respectively, based on comparisons of the Ca^2+^ sufficient and deficient treatments (Fig. 1a). In kernels, 364 genes were up regulated and 791 genes were down regulated with Ca^2+^ sufficient comparing to Ca^2+^ deficient treatment. In shells, the number of up-regulated gene was 3134, while the number of down regulated gene was 1489 upon Ca^2+^ sufficient to deficient treatment. However, the gene expression patterns of the majority of DEGs differed between kernels and shells. For example, among 3134 up-regulated genes in shells, only 70 genes were also induced in kernels. Noticeably, several genes showed the opposite expression trend between kernels and shells. For example, 287 genes were up-regulated in shells, but down-regulated in kernels. Little genes showed similar expression trends in kernels and shells; in both tissues (Fig. 1b).

Histological staining was also conducted to assess the differences in shell development. The shape and arrangement of shell cells under Ca^2+^ sufficient treatment were uniform, with more lignin in the middle-layer stereid bands than in shell cells under the Ca^2+^ deficient treatment (Fig. 1c).

### GO and KEGG enrichment of DEGs

**Figure 2:**
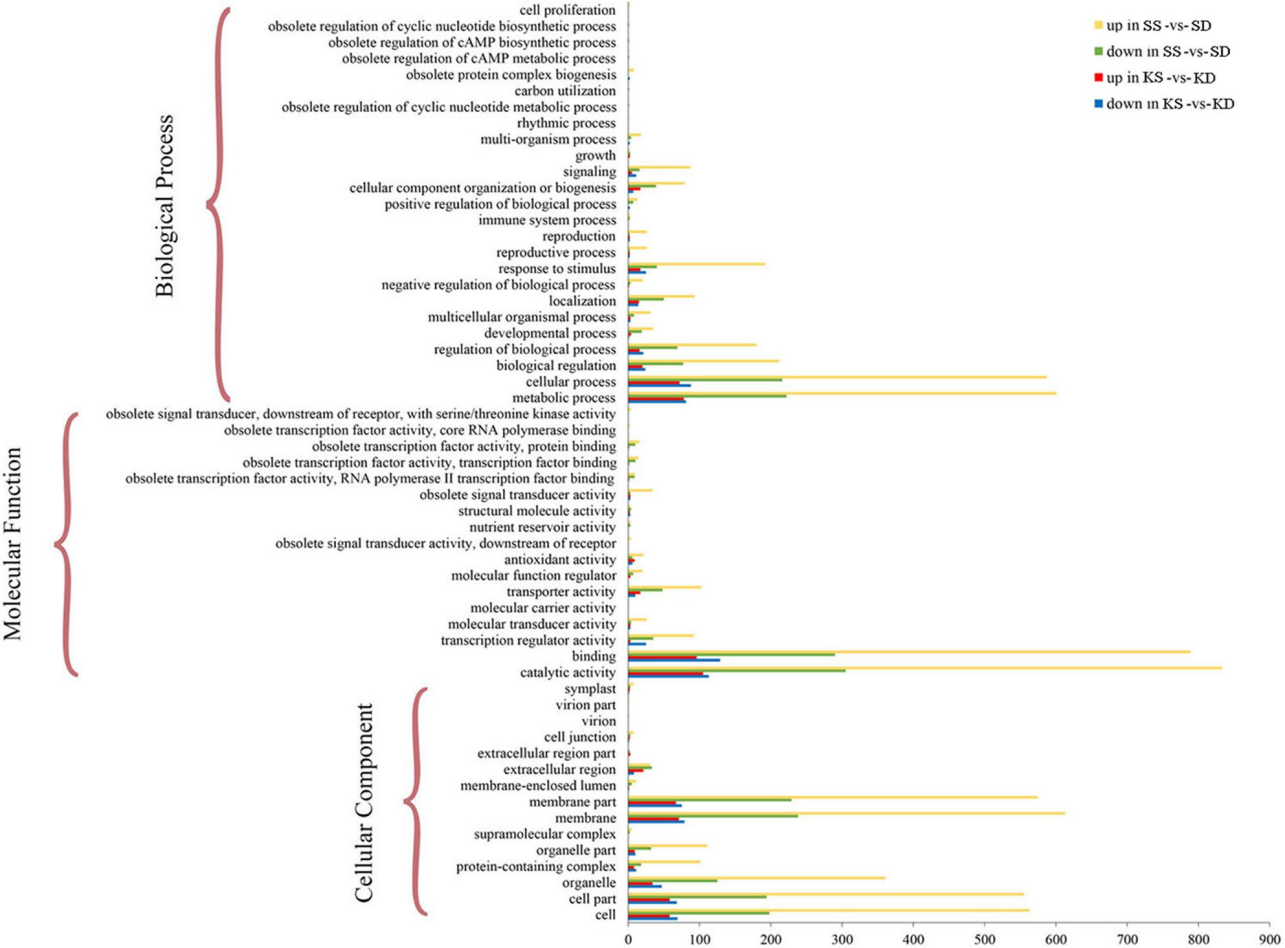
GO analysis of differently expressed genes. Biological process, cellular component and molecular function were three main categories. The x-axis indicates the number of genes in a category, and the y-axis indicated the GO terms.

Using Blast2GO, DEGs identified in kernels and shells under Ca^2+^ sufficient and deficient treatments were assigned to Gene Ontology (GO) terms. GO enrichment analysis indicated that 1145 and 2637 GO terms were enriched in kernel and shell tissues, respectively. Then the data were visualized using the WEGO2.0 web-based tool, according to three main categories: cellular component, molecular function, and biological process (Fig. 2). In the cellular component category, DEGs identified in kernel and shell tissues were enriched in four main terms, “cell,” “cell part,” “membrane,” and “membrane part.” These results emphasize the potential contribution of cells and membranes to the process of peanut pod development in response to Ca^2+^ content. The most abundant molecular function terms were “catalytic activity” and “binding.” For biological processes, “metabolic process,” “cellular process,” “biological regulation,” “regulation of biological process,” and “response to stimulus” were the five top categories, implying that metabolic activity changed more between Ca^2+^ treatments.

**Figure 3:**
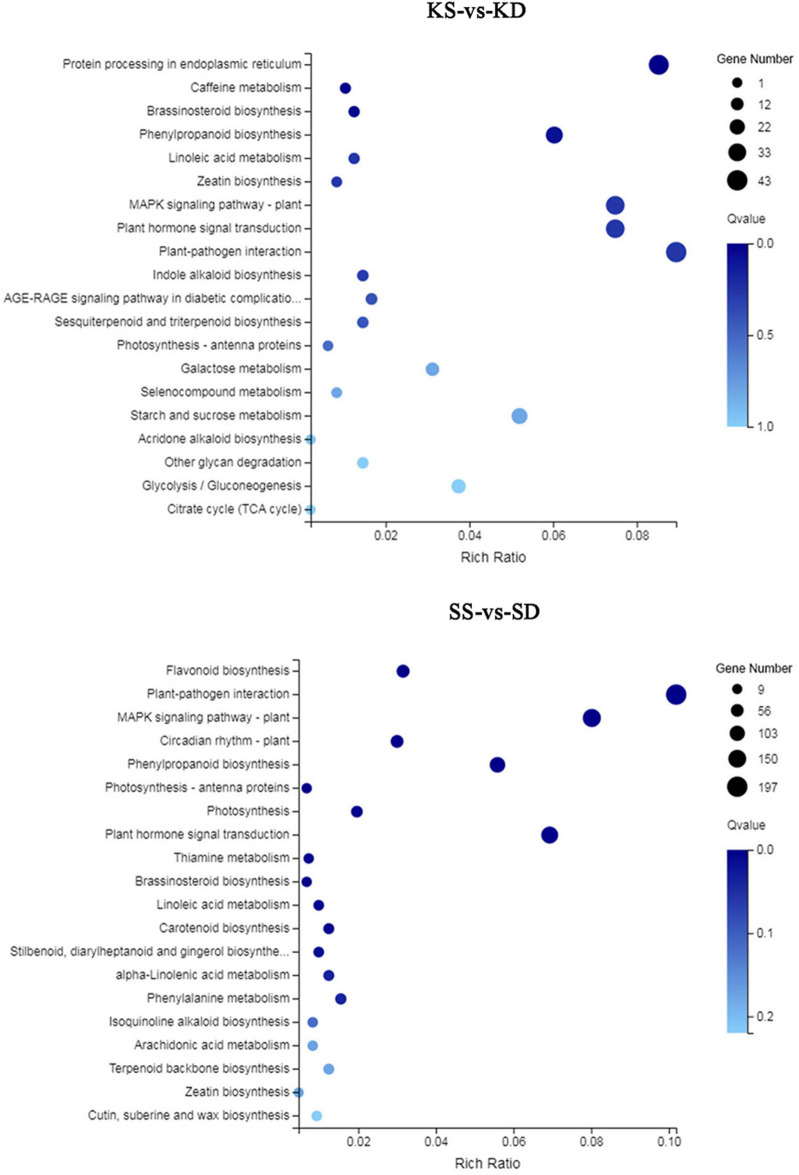
KEGG pathway analysis of differently expressed genes.

To elucidate the molecular mechanisms and regulatory networks underlying peanut pod development with different Ca^2+^ treatments, KEGG enrichment pathway analysis was also conducted. Twenty KEGG pathways were enriched in kernel and shell tissues, respectively (Fig. 3). In kernels, DEGs were mainly enriched for “Protein processing in endoplasmic reticulum,” “Phenylpropanoid biosynthesis,” “MAPK signaling pathway-plant,” “Plant hormone signal transduction,” and “Plant-pathogen interaction.” DEGs in kernels were also enriched for “Caffeine metabolism,” “Brassinosteroid biosynthesis,” “Linoleic acid metabolism,” and “Zeatin biosynthesis,” suggesting free Ca^2+^ participates in the synthesis of many kinds of substances and many metabolic processes.

In shells, the mainly KEGG pathways enriched among DEGs were very similar to those observed in kernels, besides “Protein processing in endoplasmic reticulum.” Other DEGs were mainly enriched in KEGG pathways that included “Flavonoid biosynthesis,” “Circadian rhythm-plant,” “Photosynthesis-antenna proteins,” “Photosynthesis,” and so on. These results suggested that DEGs in shell tissues participated in different biological processes compared to DEGs in kernels.

**Figure 4:**
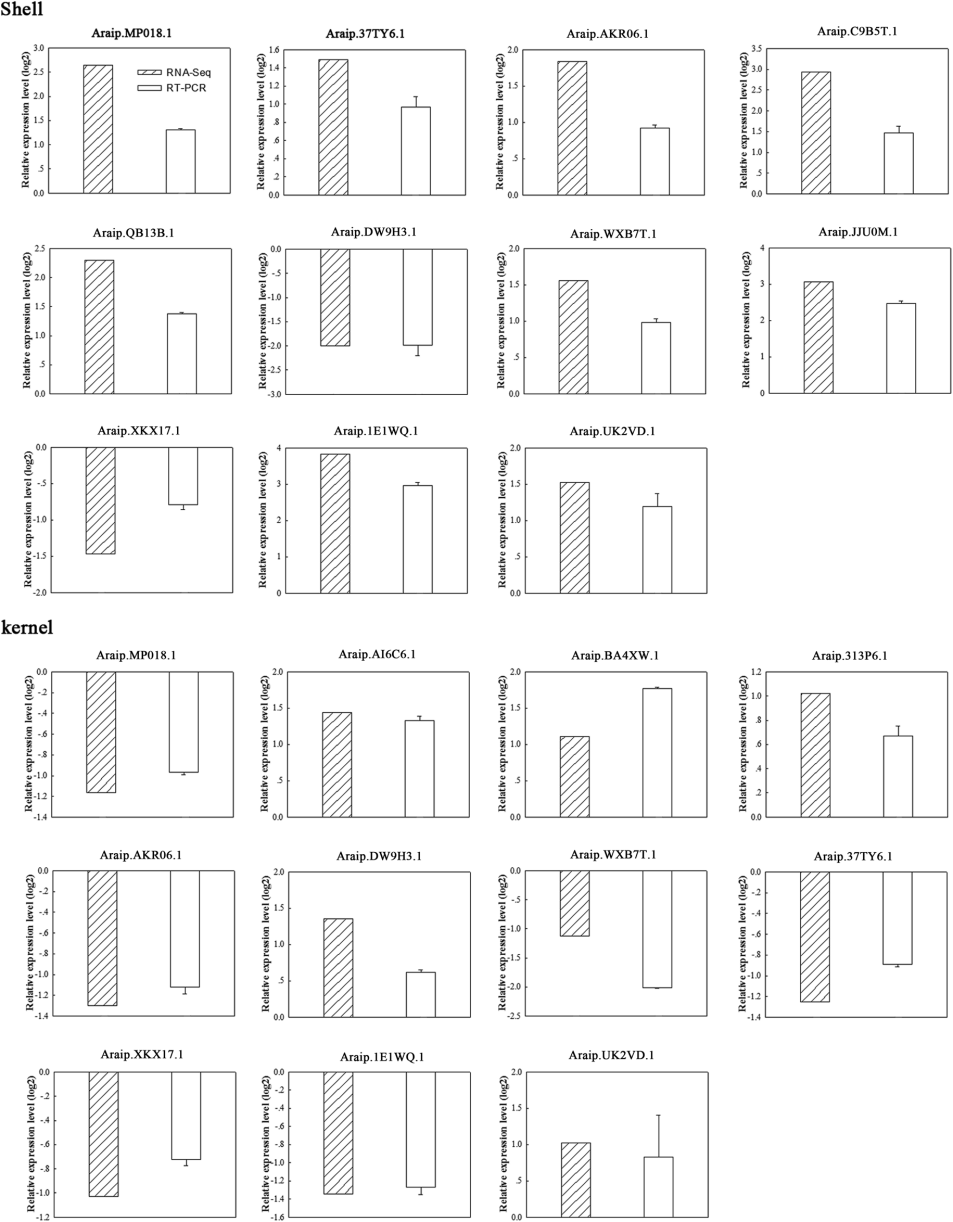
qRT-PCR verification of expression of the selected genes. Error bars indicate SE obtained from three biological replicates.

To validate the expression levels estimated from the transcriptomic data, qRT-PCR of 22 DEGs selecting from KS, KD, SS, and SD samples was conducted (Fig. 4). The consistency between the RNA-seq and the qRT-PCR data confirmed the reliability of the transcriptome sequencing results.

### Ca^2+^related DEGs in peanut kernels and shells under Ca^2+^ deficient condition

Under sufficient and deficient Ca^2+^ treatments, 1155 and 6423 DEGs in total were detected in kernels and shells, respectively. According to NR annotation, genes involved in Ca^2+^ signal transduction and responses were identified. In eukaryotes, calmodulin is the main Ca^2+^ sensor, and it can bind Ca^2+^ and regulate the activity of many proteins in response to Ca^2+^ signals. In peanut kernels, one gene encoding calmodulin protein and two genes encoding calmodulin-binding proteins were down-regulated under the sufficient Ca^2+^ treatment compared to the Ca^2+^ deficiency treatment. In shells under exogenous Ca^2+^ treatment, three genes encoding calmodulin were up-regulated. The expression of fourteen genes encoding calmodulin-binding protein changed, and among them, twelve and two genes were up-regulated and down-regulated, respectively, under the Ca^2+^ sufficient treatment relative to the Ca^2+^ deficient treatment. The expression of three Ca^2+^-transporting ATPase genes, three calcium-binding protein genes, and one calcineurin B gene were lower in kernels under the Ca^2+^ sufficient treatment. However, five Ca^2+^-transporting ATPase genes and six calcium-binding protein genes showed different expression levels in shells. Two genes encoding calcineurin B showed a similar expression trend in shells. Additionally, one calreticulin gene, five Ca^2+^-dependent protein kinase genes, three Ca^2+^ uniporter protein genes, one Ca^2+^ permeable stress-gated cation channel gene, and two cation/calcium exchanger genes were up-regulated while one cation/calcium exchanger gene was down-regulated in SS compared to SD. The relative abundance of Ca^2+^ binding and signal transduction related genes changing in peanut shells suggests that shells may be more sensitive to Ca^2+^ than that in kernels.

The IQ domain-containing protein was thought to be a novel calmodulin-binding protein that participates in multiple Ca^2+^ regulating physiological processes. Three genes encoding IQ domain-containing protein were determined to be DEGs in kernels, and their expression was lower under Ca^2+^ sufficient conditions. Among the twelve differentially expressed IQ-DOMAIN genes, eight and four were up-regulated and down-regulated, respectively, in SS compared to SD. Chitinase proteins play an important role in altering root systems under multiple environmental conditions through Ca^2+^ signaling^16^. Two chitinase genes exhibited changed expression levels between the KS and KD, although the trends in their expression differed. In SS samples, three genes encoding glutamate decarboxylase, which binds to calmodulin, were up-regulated, while five calcium-dependent protein kinase genes, which are involved in Ca^2+^ signal transduction in plants, three glutamate decarboxylases, and one calmodulin (CaM) binding protein were up-regulated in SS compared to SD. These results suggest Ca^2+^ deficiency in soil affected the Ca^2+^ signal response and transduction in peanut kernels and shells.

### Plant hormone related DEGs in peanut kernels and shells under Ca^2+^ deficient condition

**Figure 5:**
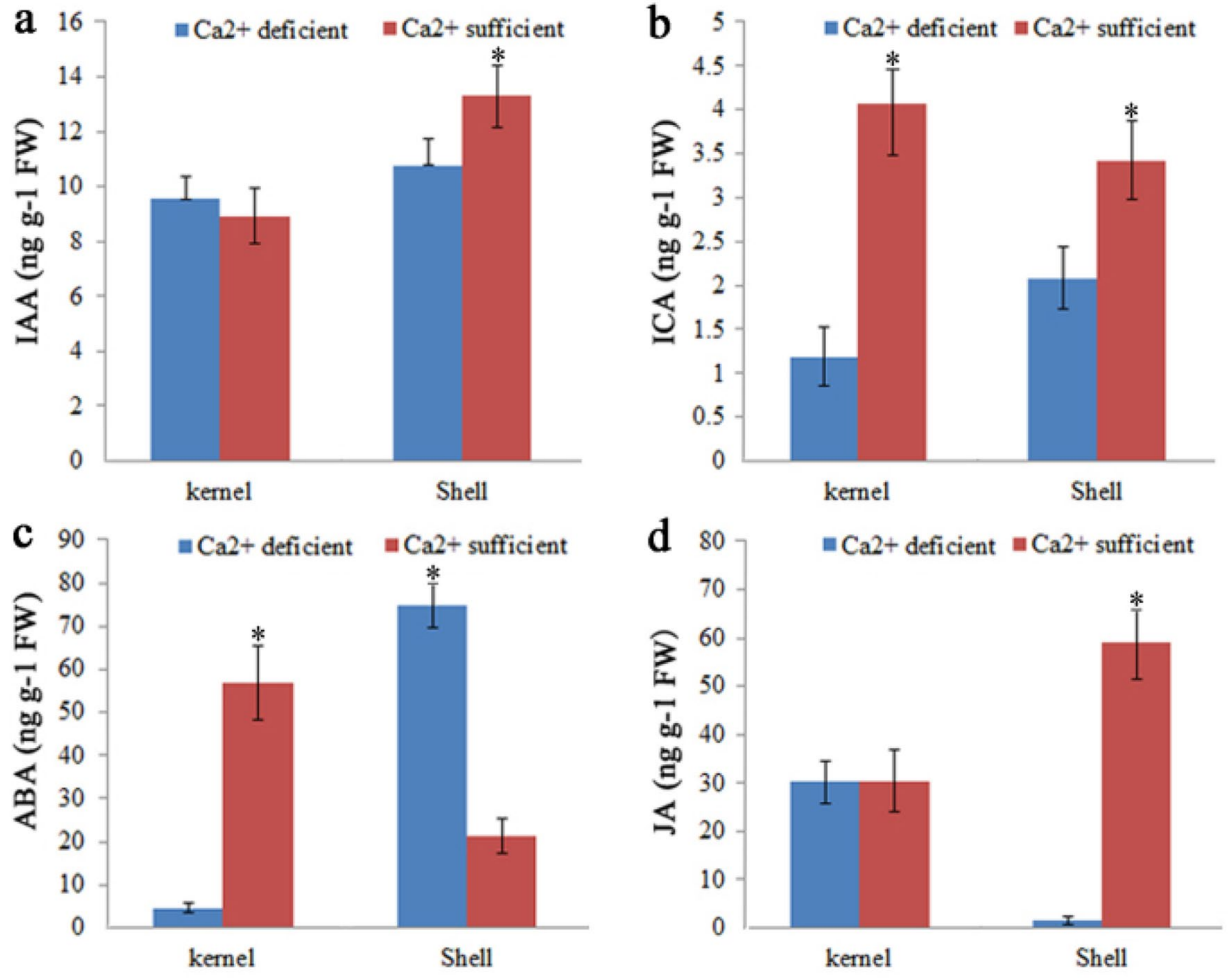
IAA, ICA, JA and ABA contents in the kernels and shells under Ca^2+^ sufficient or deficient treatments.

Figure 5a,b showed that the indole-3-acetic acid (IAA) content was significantly higher in SS than SD. Several plant hormone metabolism- and signal transduction-related genes were also differentially expressed in kernels and shells between Ca^2+^ deficiency and sufficiency treatments. Indole-3-acetic acid-amido synthetase catalyzed the formation of amino acid conjugates to modulate levels of active auxins in plants. Two genes encoding this enzyme were both down-regulated in KS relative to KD, while four indole-3-acetic acid-amido synthetase genes were up-regulated in SS compared with SD. Genes encoding flavin-containing monooxygenase, which regulates auxin biosynthesis in plants, were up-regulated in KS and SS. More genes involved in auxin biosynthesis and signal transduction were detected in SS than in SD. One gene encoding tryptophan aminotransferase-related protein, three genes encoding indole-3-pyrunate monooxygenase YUCCA, and six genes encoding IAA-amino acid hydrolase ILR1 were up-regulated in SS compared to SD. Six auxin response factor genes, three auxin-induced genes, two auxin transporter genes, and one PINOID gene were all up-regulated in SS compared to SD. These results suggest that more auxin-related DEGs were detected up-regulated in shells than in kernels.

Three kinds of GA biosynthesis enzymes were identified differentially expressed in kernels, while more kinds of GA biosynthesis and GA responsive proteins were detected in shells. Two genes encoding ent-copalyl diphosphate synthase showed down-regulation in KS. However, one ent-kaurenoic acid oxidase gene and one gibberellin 20 oxidase gene were up-regulated in KS compared to KD. In shells, four genes encoding gibberellin 20-oxidase were up-regulated under Ca^2+^ sufficient treatment compared to Ca^2+^ deficient treatment. In KS, two genes encoding chitin-inducible gibberellin (GA)-responsive protein were down-regulated compared to that in deficient conditions, while two chitin-inducible gibberellin-responsive protein genes showed the opposite expression trend in shells. Compared with the Ca^2+^ sufficient treatment, one DELLA gene was down-regulated in shells under the Ca^2+^ sufficient treatment. One MYB62 transcription factor gene, which regulates gibberellic acid biosynthesis, was up-regulated, and one SHI RELATED SEQUENCE gene, which represses gibberellin response, was down-regulated in shells under Ca^2+^ sufficient treatment compared to the Ca^2+^ deficient treatment. Additionally, two genes encoding gibberellin-regulated proteins and five genes encoding gibberellin 2-beta-dioxygenase showed differential expression in SS compared to SD, and their expression trends were dissimilar.

Cytokinin hydroxylase catalyzes trans-zeatin biosynthesis, and cytokinin dehydrogenase catalyzes cytokinin degradation. In KS, two genes encoding cytokinin hydroxylase were down-regulated and two genes encoding cytokinin dehydrogenase were up-regulated compared to Ca^2+^ deficient conditions. In shells, one cytokinin hydroxylase gene showed the opposite expression trend, and three cytokinin dehydrogenase genes showed the same expression trend compared with the kernels. One zeatin *O*-glucosyltransferase gene was down-regulated, and four genes encoding cytokinin riboside 5′-monophosphate phosphoribohydrolase were up-regulated in SS compared to SD. Two genes encoding adenylate isopentenyltransferase exhibited changed expression levels in SS; one was up-regulated, while the other one were down-regulated. Two phosphatase 2C genes, one C2-domain ABA-related gene, one abscisic acid receptor PYL11 gene, and one abscisic acid 8′-hydroxylase gene were all down-regulated in KS compared with KD. In SS, five short-chain dehydrogenase reductase genes, three EARLY-RESPONSIVE TO DEHYDRATION (ERD) genes (which act as negative regulators of abscisic acid responses), four C2-DOMAIN ABA-RELATED genes, and five abscisic acid 8′-hydroxylase genes were up-regulated compared to the Ca^2+^ deficient treatment. It was reported that overexpression of ERD15 reduced the ABA sensitivity of *Arabidopsis*, and abscisic acid (ABA) 8′-hydroxylase catalyzes the first step in the oxidative degradation of ABA^17,18^. Thus, the lower ABA content in SS was likely associated with the up-regulated expression of these genes (Fig. 5c).

Two genes encoding ethylene-responsive transcription factor, one gene encoding 1-aminocyclopropane-1-carboylate synthase, and one gene encoding 1-aminocyclopropane-1-carboxylate oxidase were all down-regulated in kernels under the Ca^2+^ sufficient condition compared to the Ca^2+^ deficient condition. Twenty-two genes encoding ethylene-responsive transcription factor changed their expression level in SS. Two genes encoding ethylene-overproduction protein were down-regulated, and one gene encoding EIN3, two genes encoding 1-aminocyclopropane-1-carboxylate synthase, and five genes encoding 1-aminocyclopropane-1-carboxylate oxidase were up-regulated in SS compared to SD. Besides the genes mentioned above, MYC2 and MYC4 transcription factors, which participate in jasmonate response, were also detected to have differential expression between Ca^2+^ sufficient and deficient conditions in peanut kernels and shells, while jasmonic acid-amido synthetase JAR1 was only up-regulated in shells. These results were in agreement with the observed increase of JA content in SS. BES1 acts as a brassinosteroid transcriptional effector; in kernels, one BES1/BZR1 gene was down-regulated, while two BES1/BZR1 genes were up-regulated in SS relative to SD. One gene encoding BRASSINOSTEROID INSENSITIVE was up-regulated in SS compared to SD.

### Development-related DEGs in peanut kernels and shells under Ca^2+^ deficient conditions

Several plant development genes were also detected in KS and KD conditions. UDP-glycosyltransferase was localized in meristem, and in kernels one gene encoding UDP-glycosyltransferase was up-regulated in Ca^2+^ sufficient condition comparing to Ca^2+^ deficient condition. One gene encoding the transcription factor RADIALIS, which belongs to the MYB transcription factor family, was up-regulated in the KS. EXORDIUM protein has been shown to promote *Arabidopsis* growth, and two EXORDIUM genes were down-regulated in kernels, while one EXORDIUM gene was up-regulated in SS. CUP-SHAPED COTYLEDON regulates postembryonic shoot meristem and organ boundary formation in *Arabidopsis*; in peanut kernels, one CUP-SHAPED COTYLEDON gene was up-regulated, while two CUP-SHAPED COTYLEDON genes were down-regulated in SS compared to SD.

Other genes involved in plant development were uniquely detected in shells. One BIG GRAIN gene, one DA1 gene, and two receptor-like protein kinase HAIKU genes, which control signaling pathways in plants, were up-regulated under the Ca^2+^ sufficient treatment compared to the Ca^2+^ deficient treatment. Several genes are required for meristem function maintenance, including TORNADO 2, TONSOKU, CLAVATA 3, BEL1-like homeodomain protein, and KINKY POLLEN. Among these genes, there were single representatives from each of the TORNADO 2, TONSOKU, CLAVATA 3, and KINKY POLLEN genes that were up-regulated, while three genes encoding BEL1-like homeodomain protein were down-regulated under Ca^2+^ sufficient conditions compared to Ca^2+^ deficient conditions. Two MADS-box transcription factor genes, two growth-regulating factor genes, and one embryo-specific protein gene showed lower expression levels under the Ca^2+^ sufficient treatment relative to the Ca^2+^ deficient treatment. Seven genes encoding LOB domain-containing protein and nine genes encoding expansin were also detected. Most of these genes were up-regulated under the Ca^2+^ sufficient treatment compared to the Ca^2+^ deficient treatment. BON1 controls plant growth homeostasis, and one BON1 gene showed higher expression under the Ca^2+^ sufficient treatment relative to the Ca^2+^ deficient treatment.

### Ion transport- and nutrient absorption-related DEGs in peanut kernels and shells under Ca^2+^ deficient conditions

Under Ca^2+^ deficient conditions, several cation absorption and transport related genes changed in expression level. One and five genes encoding zinc transporters were down-regulated in kernels and shells under the Ca^2+^ sufficient treatment compared to the Ca^2+^ deficient treatment. One aluminum-activated malate transporter gene was up-regulated under the Ca^2+^ sufficient treatments compared to the Ca^2+^ deficient treatment. STE20/SPS1-related proline-alanine-rich protein kinase is critical for sodium absorption; one gene encoding this protein and one sodium/calcium exchanger NCL were up-regulated under the Ca^2+^ sufficient treatment compared to the Ca^2+^ deficient treatment. Two genes encoding potassium transporter 5 and one gene encoding magnesium transporter NIPA1 were up-regulated in SS compared to SD. Transcripts of nodulin 26, an ion channel protein, were up-regulated under the Ca^2+^ sufficient treatment. In KS, the expression level of cation/H^+^ antiporter was up-regulated relative to that in KD. In kernels, transcription factor bHLH100, which controls iron homeostasis, and one bHLH100 gene was up-regulated in kernels under the Ca^2+^ sufficient treatment compared to the Ca^2+^ deficient treatment. In shells, three genes encoding cyclic nucleotide-gated ion channel protein and one gene encoding mechanosensitive ion channel protein were up-regulated under the Ca^2+^ sufficient treatment compared to the Ca^2+^ deficient treatment.

Nitrate and phosphate absorption-related genes were also detected in peanut kernels. The expression levels of three genes encoding NRT1/PTR family members changed, among them, two genes were up-regulated, and one gene was down-regulated under Ca^2+^ sufficient conditions compared to Ca^2+^ deficient conditions. One gene encoding a high affinity nitrate transporter was down-regulated under the Ca^2+^ sufficient treatment compared to the Ca^2+^ deficient treatment. In shells under the Ca^2+^ sufficient treatment, one gene encoding transcription factor HRS1, which integrates nitrate and phosphate signals, was up-regulated compared to the Ca^2+^ deficient treatment. SPX domain-containing proteins participate in phosphate homeostasis. Three genes encoding this protein were differentially expressed in shells between the different Ca^2+^ treatments. Two SPX domain-containing protein genes were up-regulated, and one was down-regulated under the Ca^2+^ sufficient treatment compared to the Ca^2+^ deficient treatment. In shells, one gene encoding vacuolar cation/proton exchanger 3 was up-regulated under the Ca^2+^ sufficient treatment compared to the Ca^2+^ deficient treatment, and two genes encoding vacuolar amino acid transporter were down-regulated.

## Discussion

Peanut is one of the most important oil and commercial crops in China, though the peanut production among different regions within China is very uneven. In the south of China, more than 1.1 million hectares were planted with peanut, however, the peanut yield per unit area was lower in this region than the national average. The red loam often found in the south of China has a lower pH, providing a weak Ca^2+^ adsorption capacity. Ca^2+^ deficiency is the main cause of empty or incompletely filled peanut pods. Peanut is a crop that needs more Ca^2+^, and during pod development more than 90% of Ca^2+^ in peanut pods is absorbed from the soil^19^. A lack of Ca^2+^ can lead to embryo abortion, empty pod formation, and massive reductions in yields.

Calcium is very important for peanut embryo and pod development^14^. Calcium is involved in maintaining the normal structure of peanut cells, and in the synthesis of multiple endogenous plant hormones. Meanwhile, endogenous plant hormones affect the absorption of calcium by peanuts^20^. In this study, the effect of calcium on gene expression of peanut kernels and shells was analyzed individually. In total, 38,894 genes were detected in peanut kernels and shells under Ca^2+^ deficient and sufficient conditions. Among them, 1151 and 6423 genes were identified as differentially expressed in kernels and shells, respectively, between calcium treatments (Fig. 1). These DEGs indicated that peanut kernels and shells showed different responses to Ca^2+^ during pod development. During peanut pod development, calcium deficiency caused many genes to change in their expression level both in kernels and shells. Based on GO and KEGG pathway analyses, DEGs identified in kernels and shells were involved in multiple physiological processes (Fig. 3). These results suggested that Ca^2+^ influences peanut pod development. Multiple Ca^2+^-binding proteins act as Ca^2+^ sensors that monitor cellular Ca^2+^ changes. Calcineurin B-like proteins (CBLs) are plant-specific Ca^2+^ sensor proteins that are similar to both the regulatory β subunit of calcineurin and neuronal calcium sensors^21^. After binding to Ca^2+^ through EF-hand motifs, CBLs specifically interact with CBL-interacting protein kinase to transmit Ca^2+^ signals^22^. The same transcription trend in Calcineurin B in both kernels and shells indicates that the response of peanut Calcineurin B involves the same signaling module under Ca^2+^ deficiency. CaM belongs to a primary and prototypical class of Ca^2+^ sensors in plant cells^23^. While CaM showed no catalytic activity, after binding to Ca^2+^ via the EF-hand motif, the configuration of CaM is changed, which exposes the high affinity binding sites in hydrophobic regions for downstream target proteins^24^. Thus, CaM functions at another level of Ca^2+^ signaling transduction to trigger specific physiological responses^25^. Besides CaM and CaM-binding protein, Ca^2+^-transporting ATPase and calcium-binding protein showed the opposite expression trend in peanut kernels and shells under the Ca^2+^ deficient treatment, indicating that the function of Ca^2+^ triggering signaling transduction in peanut shells was greater than that in peanut kernels under Ca^2+^ sufficient conditions.

Many kinds of plant hormones play multiple functions during peanut pod development^26,27^. Under different Ca^2+^ treatments, the expression trends of several genes involved in phytohormone biosynthesis and signal transduction were changed. In plants, indole-3-acetic acid-amido synthetase reduced the levels of active auxin to formation amino acid conjugates. However, the auxin biosynthesis regulating gene flavin-containing monooxygenase showed differential expression in peanut kernels between the Ca^2+^ treatments. In peanut shells, the expression of more genes involved in auxin signal transduction and biosynthesis changed. These results suggest that Ca^2+^ deficiency in the soil substantially influences auxin homeostasis. Other genes related to plant hormones, such as GA, cytokinin, and ethylene, as well as regulatory and signal transduction-related genes also exhibited differential expression^27^. Previous studies have reported that phytohormones, including auxin, ethylene and gibberellins, play important roles in the early development of peanut pods^3^. Auxin is necessary for grain size and starch accumulation, and GA, combined with calcium and magnesium, can affect grain weight and seed yields^28^. These changes in plant hormone balance induced by Ca^2+^ deficiency in soil can further transmit signals to affect the development of peanut pods.

Several development-related genes were also identified in the peanut tissues assayed under different Ca^2+^ treatments. In peanut shells, UDP-glycosyltransferase, RADIALIS, and CUP-SHAPED COTYLEDON showed higher expression levels under the Ca^2+^ sufficient treatment compared to the Ca^2+^ deficient treatment. High UDP-glycosyltransferase expression levels markedly promote growth and development of transgenic alfalfa^29^. RADIALIS and CUP-SHAPED COTYLEDON genes play an important role in plant early morphogenesis^30–32^. In peanut shells, EXORDIUM, which is known as a brassinosteroid-regulated gene that controls plant growth and development^33^_,_ was highly expressed under the Ca^2+^ sufficient treatment. DA1, TRANSPARENT TESTA 1, TORNADO 2, TONSOKU, CLAVATA 3, BEL1-like homeodomain protein, KINKY POLLEN, MADS-box transcription factor, LOB domain-containing protein, and expansin also participate in plant growth and development processes^34–40^. Combined with the significant difference in shell phenotypes between Ca^2+^ sufficient and deficient conditions (Fig. 1c), these results indicate that Ca^2+^ plays an important role in regulating peanut shell formation and development.

Calcium acts as a plant nutrient, and it maintains the membrane stability and integrity of cells. Ca^2+^ required for the development of peanut pods is absorbed not only by the elongated peg but also directly from the surrounding soil medium. Ca^2+^ deficiency in soil can induce ions disorder. The expression levels of genes encoding zinc transporters, potassium transporters, magnesium transporters, aluminum-activated malate transporters, sodium/calcium exchangers, sodium transporters, and cation/H^+^ antiporters were changed in accord with the expression of positive ion transporters under different Ca^2+^ treatments. These results suggested that Ca^2+^ deficiency influenced ion homeostasis in cells and impeded nutrient absorption. This phenomenon indicated that Ca^2+^ signal transduction and lignification were enhanced under Ca^2+^ sufficient conditions, which enables the transmission of nutrients from both the aerial peg and the soil to the shell, with little influence on seed kernels.

**Figure 6:**
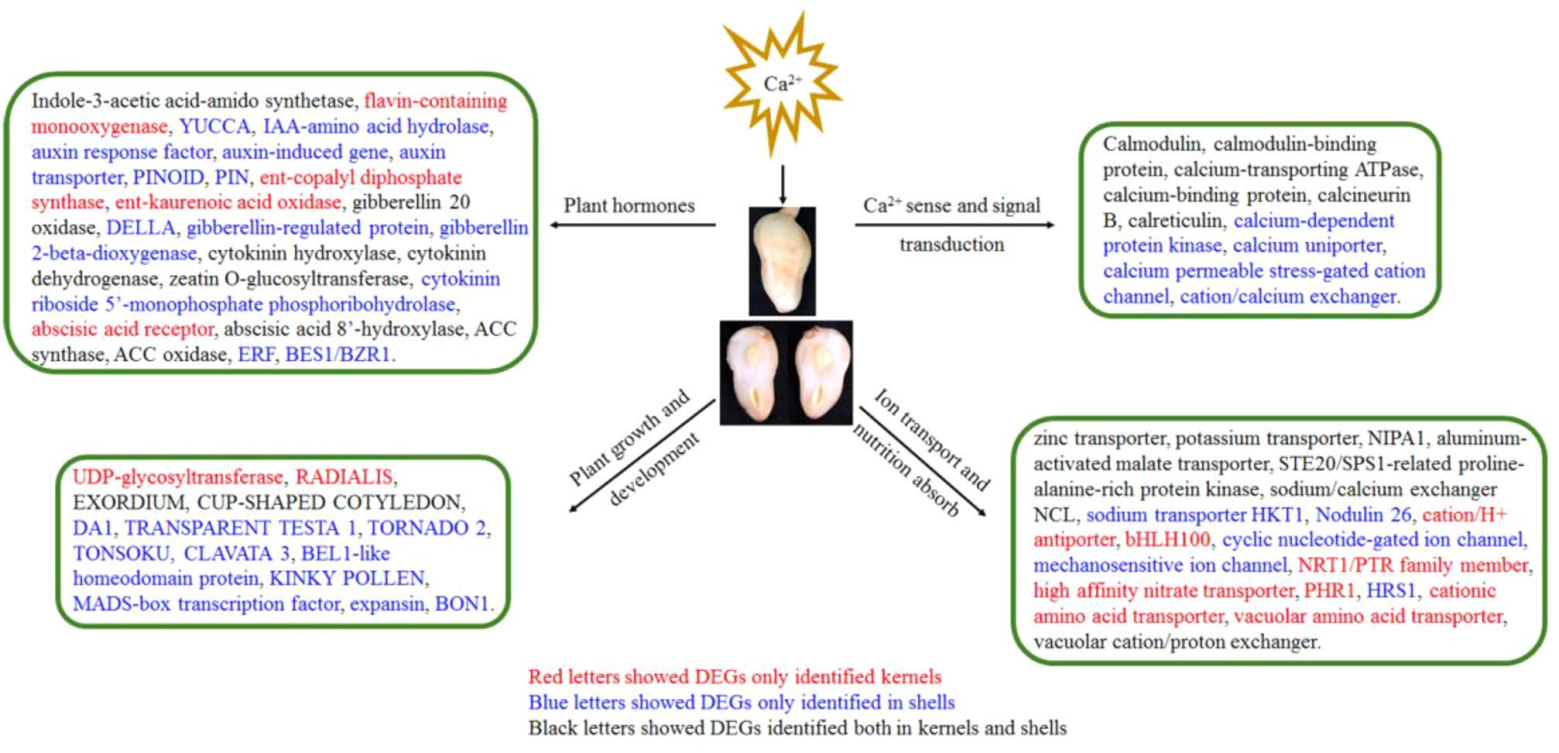
Summarized gene regulation networks of DEGs in peanut kernels and shells.

In conclusion, changes in hormones, development, and nutrition absorption-related genes under sufficient Ca^2+^ conditions further elucidated the crucial mechanistic role of Ca^2+^ on peanut pod development (Fig. 6). In the early stage of pod development, Ca^2+^ first promoted shell development and nutrient absorption, on the same time, less of an effect on the formation of seed kernels was observed. The well-developed shells may play a key role in seed kernel filling, and we will further study the mechanism by which Ca^2+^ deficiency induces pod abortion at later stages of pod development.

## Material and methods

### Plant materials and growth conditions

Cultivated peanut (HuaYu 22) were grown in the farm of Shandong Academy of Agricultural Sciences in early May of 2018. Three seeds were dispersedly sown in one flowerpot with 60 cm in diameter. Laterite from Hunan Province of China which has low content of exchangeable calcium (0.74 cmol/kg) was used as the free Ca^2+^ deficient treatment. Laterite treated with 50 kg plaster (CaO) per 667 m^2^ was used for the free Ca^2+^ sufficiency treatment. The plaster was applied before sowing. During the growth and development of peanut, 15 days after pegging (DAP) was confirmed to be the most sensitive period for the free Ca^2+^ content in the soil. At this period, the pods were collected and manually diveded into two parts, namely shells and kernels. Then, shells and kernels from the free Ca^2+^ deficient treatment and free Ca^2+^ sufficient treatment were collected separately, frozen immediately in liquid nitrogen, and stored at − 80 °C for the subsequent experiments. For both shells and kernels, three biological replicates were prepared.

### RNA isolation, cDNA library construction, and sequencing

Total RNA was isolated from shells and kernels using Trizol reagent according to the manufacturer’s instructions. RNA quality and purity were detected using Agilent 2100 (Agilent Technologies, Santa Clara, CA, USA) and NanoDrop platforms, respectively. The isolated mRNA was enriched with Oligo (dT) beads and segmented with interrupt buffer, followed by reverse transcription using random N6 primers. The second cDNA strand was synthesized in a reaction system containing buffer, dNTPs, RNase H, and DNA polymerase. The synthesized double-stranded DNA ends were purified. End repair and 3′-end single nucleotide A (adenine) addition were performed. Then, sequencing adaptors were added to the fragments and the products were amplified by PCR with specific primers. Finally, the cDNA libraries were sequenced using the Illumina HiSeq 4000 platform. Whole dataset has been uploaded in NCBI Sequence Read Archive with accession number SRP167771.

### Paraffin section

Only the shells were used as sample tissues for sectioning assay, and they were fixed with fixative solution for more than 24 h. The tissue was taken out and flattened using the scalpel. Put the repaired tissue and the corresponding label in the dehydrated box for dehydration. The tissue was embedded in paraffin and cut with a microtome blade to a 4-μm thickness. Samples were stained with safranin O/fast green and visualized with a Nikon ECLIPSE TI-SR microscope.

### Data analysis

The raw data were collected after sequencing. First, reads with low quality, joint contamination and high content of unknown base N were filtered out using SOAPnuke (− l 15 − q 0.2 − n 0.05)^41^. The filtered data were hereafter referred to as clean reads and were thus used for further bioinformatics analysis. Clean reads were then compared to the reference genome and reference gene sequences using HISAT2 (– phred64 – sensitive – no-discordant – no-mixed − I 1 − X 1000)^42^. and Bowtie2 (− q – phred64 – sensitive – dpad 0 – gbar 99999999 – mp 1,1 – np 1 – score-min L,0,− 0.1 − p 16 − k 200)^43^ respectively. StringTie (− f 0.3 − j 3 − c 5 − g 100 − s 10,000 − p 8) was used to reconstruct the transcript of each sample, Cuffmerge (− p 12) was used to integrate the reconstruction information of all samples and compare the integrated transcripts with the reference annotation information. Transcripts of class code type ‘u’ (i.e., unknown transcripts belonging to intergenic regions), ‘I’ (i.e., transcripts located in known intron regions of genes), ‘o’ (i.e., transcripts that intersect with exons of known genes), and ‘j’ (i.e., potential new transcripts or fragments, with at least one junction site consistent with the reference gene) were selected and defined as new transcripts. CPC (the default parameters) was used to predict the potential of protein coding for new transcripts. Finally, new transcripts that potentially code for proteins were added to the reference gene sequence to obtain complete reference sequence information, which was used for subsequent analysis. According to the reference genome comparison results, GATK was used to detect SNP and InDel information of each sample, mainly focusing on mutation detection and genotyping^44^. Using RNA-seq Expectation Maximization (RSEM) software, gene quantification was performed which maximum likehood abundance was to estimate the statistical model, including the modeling of paired-end (PE) and variable-length reads, fragment length distributions, and quality scores, and to determine which transcripts were isoforms of the same gene^45^. Fragments per kilobase of exon per million fragments mapped (FPKM) was used to calculate the gene expression level of each sample as follows: FPKM = [10^6^C/(NL/10^3^)]. In the formula above, FPKM was the expression of gene A, C is the number of fragments uniquely aligned to gene A, N is the total number of fragments uniquely aligned to all genes, and L is the number of bases on gene A^46^. Cor function in R software was used to calculate Pearson correlation coefficent between every two samples. DEGseq method was based on poisson distribution, and differently expressed gene (DEG) detection was performed according to the method described in Wang^47^. In order to improve the accuracy of DEGs, genes were defined with a difference multiple of more than twice and a q-value ≤ 0.001, and screened them as significantly DEGs. According to the GO and KEGG annotation results and official classification, we classified the function of the DEGs, and used phyper function in R software for enrichment analysis. The calculation method of p-value was as follows:

$${\text{P}} = 1 - \sum\limits_{i = 0}^{m - 1} {\frac{{\left( {\begin{array}{*{20}c} M \\ i \\ \end{array} } \right)\left( {\begin{array}{*{20}c} {N - M} \\ {n - i} \\ \end{array} } \right)}}{{\left( {\begin{array}{*{20}c} N \\ n \\ \end{array} } \right)}}}$$

The p-value was FDR corrected, and the function of FDR ≤ 0.01 was generally considered as significant enrichment. For the identification of transcription factors, getorf (− minsize 150) was used to detect the ORF of Unigene, then hmmsearch (the default parameter) was used to compare ORF to the structural domain of transcription factor protein, and then the ability of unigene was identified according to the transcription factor family characteristics described by PlantTFDB.

### qRT-PCR analysis

Peanut pod samples under the same treatment as those in RNA-seq were validated using qRT-PCR. 15 peanut plants from 5 flowerpots in each treatment were selected randomly, and the shells and kernels of the plants were sampled separately. Three biological replicates were used to calculate the relative expression. First, cDNAs were reverse-transcribed using the first-strand cDNA synthesis kit (TAKARA, Dalian, China). The primers sequences were designed with Primer 5 software (listed in Supplemental Table S1). Each reaction mixture included 10 μl 2 × FastStart Universal SYBR Green Master Mix, 0.5 μl primer (10 mM), and 70 ng template cDNA, for a volume of 20 μl. The ABI 7500 platform was used as the qRT-PCR amplification instrument. *Tua-F* and *Tua-R* were used as the control genes to normalize the expression level. The 2^−ΔΔCT^ method was used to calculate the relative expression level^48^.

### Hormone determination

50 mg samples were ground to powder using MM 400 (Retsch) and extracted with methanol: water: formic acid = 15:4:1 (v:v:v). After concentration, the samples were dissolved in 100 μl 80% methanol–water solution and filtered through 0.22 μm PTFE membrane. The samples were placed in the injection bottle for LC–MS/MS analysis, which was determined by commercial service provider (Metware Biotechnology, Co., Ltd. Wuhan, China). JAs (MEJA, JA, H2JA and JA-ILE), CK (IP, tZ, cZ and DZ), auxin (IAA, ME-IAA and ICA) and ABA contents were detected based on the AB Sciex QTRAP 6500 LC–MS/MS platform. Three replicates of each assay were performed.

## Supplementary information

Supplementary Information 1.

Supplementary Information 2.

Supplementary Figure S1.

Supplementary Figure S2.

Supplementary Figure S3.

Supplementary Figure S4.

Supplementary Figure S5.

Supplementary Figure S6.

Supplementary Figure S7.

Supplementary Table S1.
