# Supplementary material for: Transcriptome of peanut kernel and shell reveals the mechanism of calcium on peanut pod development
Source: Sci Rep. 2020 Sep 24;10:15723. doi: 10.1038/s41598-020-72893-9 (PMC7518428; doi:10.1038/s41598-020-72893-9)
Supplement: Supplementary file 1 — Supplementary Information 1. [file 41598_2020_72893_MOESM1_ESM.docx]

**Supplementary Materials:**

Fig. S1 Random analysis of sequencing.

Fig. S2 Gene coverage analysis.

Fig. S3 Sequencing saturation analysis.

Fig. S4 Statistical results of 5 variable splicing events.

Fig. S5 Statistical results of 6 types of SNP.

Fig. S6 Distribution of SNP sites in genome.

Fig. S7 Distribution of InDel in genome.
