# Supplementary material for: Transcriptome of peanut kernel and shell reveals the mechanism of calcium on peanut pod development
Source: Sci Rep. 2020 Sep 24;10:15723. doi: 10.1038/s41598-020-72893-9 (PMC7518428; doi:10.1038/s41598-020-72893-9)
Supplement: Supplementary file 2 — Supplementary Information 2. [file 41598_2020_72893_MOESM2_ESM.docx]

R software attribution is ensured at <https://pan.genomics.cn/ucoffice/> pdfViewer/viewer.html?officeDesc=RNAref%28%E6%9C%89%E7%94%9F%E7%89%A9%E5%AD%A6%E9%87%8D%E5%A4%8D%29++-Dr+Tom+II%E4%BA%A4%E4%BB%98.pdf&file=https%3A%2F%2Fpan.genomics.cn%2Fucoffice%2Fapi%2Ffile%2FpreviewsStream%3Fkey%3D8F364185A37AE0C1DFDB35B9A7F67D565B70C6845F9E1A377D2F0B71BB3932F9E6006AAE265797FA7B1DB84F302CB5C935E47E623215AF9F08861798A2C24203FE3FE84808D58C59B2CDD3B0B46583A51F5C879646E944C3C2587523DD61BAFEAD1F495CB2B32BA77B4D7A713642C09E2D16F60B0EBA438A26E0269F0F2506404F3382FB10DC1259CF5242FDA42CFDE3C09D89AF55634741917BBBC521F528EFC84CD20F8B9FB0C10688A3771AFD057AD07636B3AF3F15A84C93F65FB6C5D9CCBD3E2231669A11ED73362805EDDC56F456908A74718FED0C0C6DAD0AF3CD499A%26path%3D%2Foutput%2Faedbe7cc98b8303c39d4bc8c24393529%2Fview.html%2Faedbe7cc98b8303c39d4bc8c24393529.
