# Supplementary figures and images for: Transcriptome of peanut kernel and shell reveals the mechanism of calcium on peanut pod development
Source: Sci Rep. 2020 Sep 24;10:15723. doi: 10.1038/s41598-020-72893-9 (PMC7518428; doi:10.1038/s41598-020-72893-9)

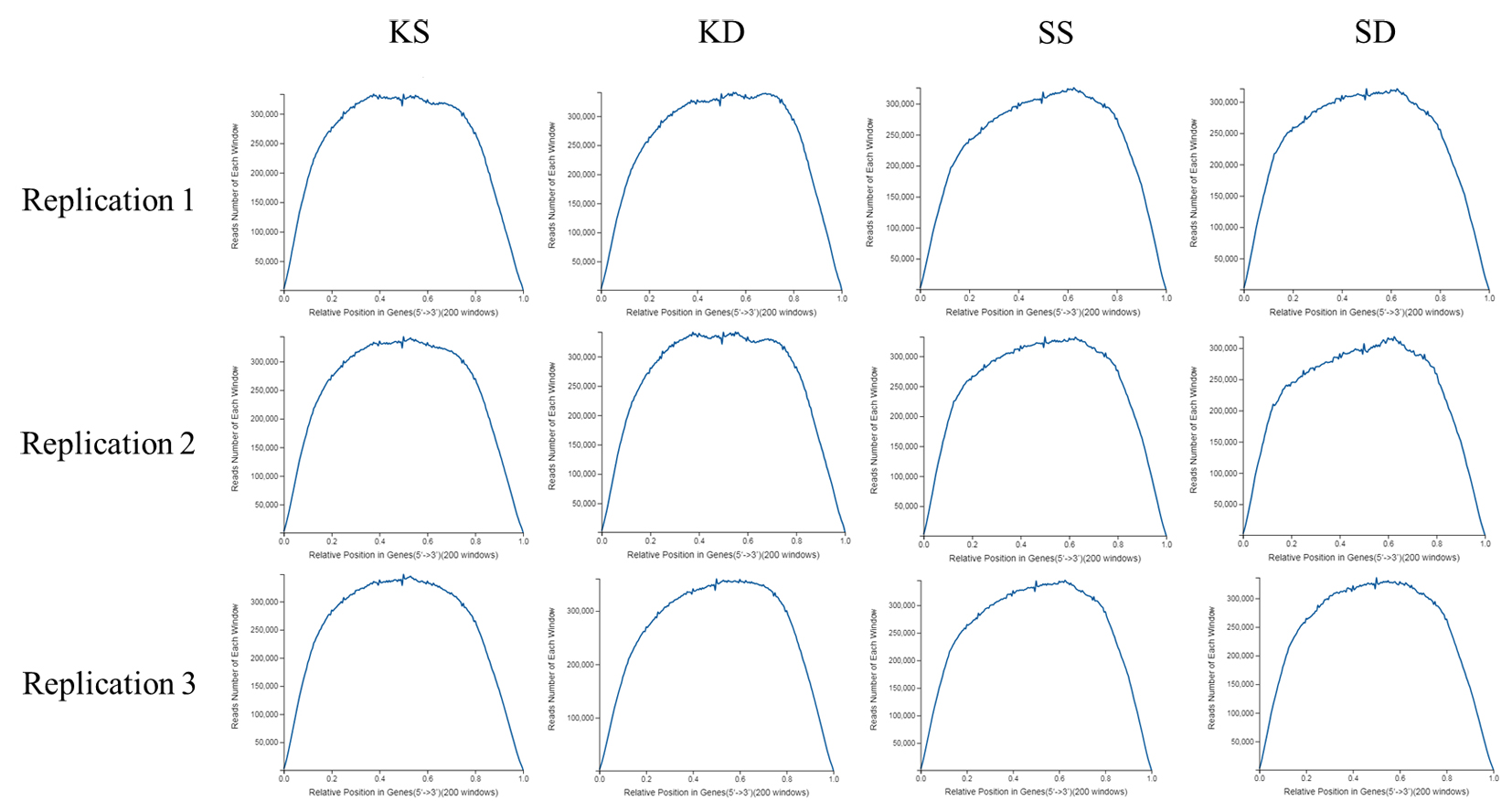

Supplement: Supplementary file 3 — Supplementary Figure S1. [file 41598_2020_72893_MOESM3_ESM.tif]

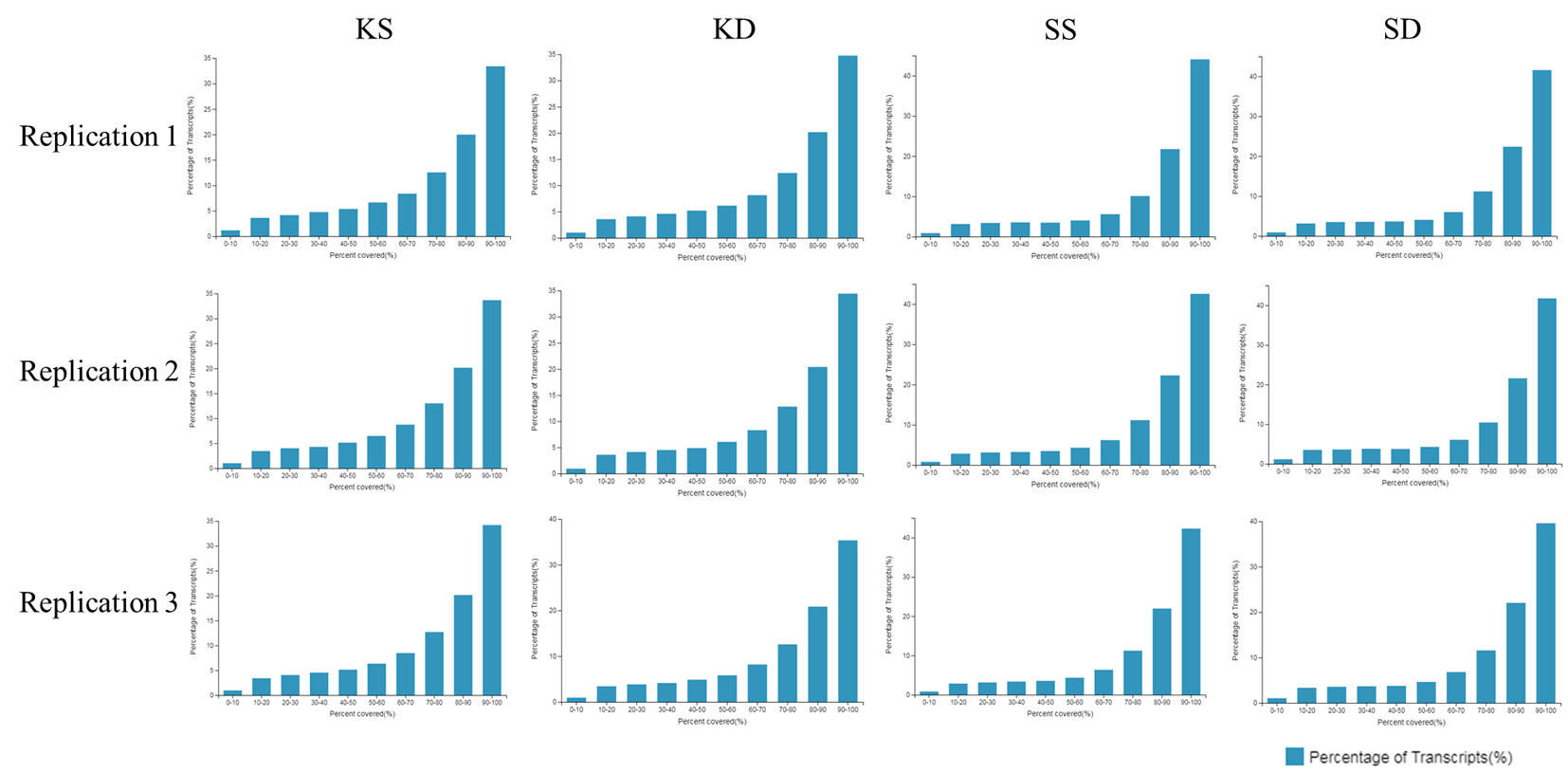

Supplement: Supplementary file 4 — Supplementary Figure S2. [file 41598_2020_72893_MOESM4_ESM.tif]

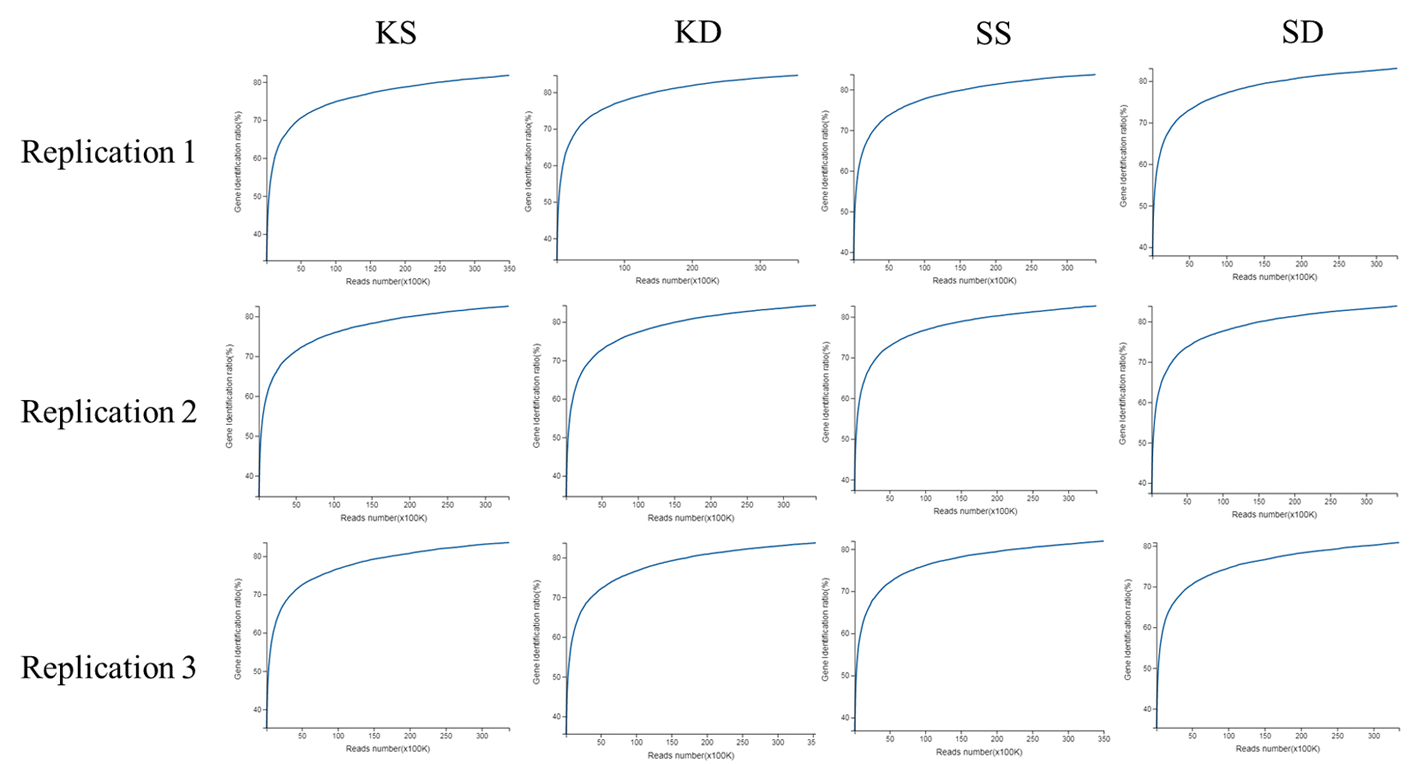

Supplement: Supplementary file 5 — Supplementary Figure S3. [file 41598_2020_72893_MOESM5_ESM.tif]

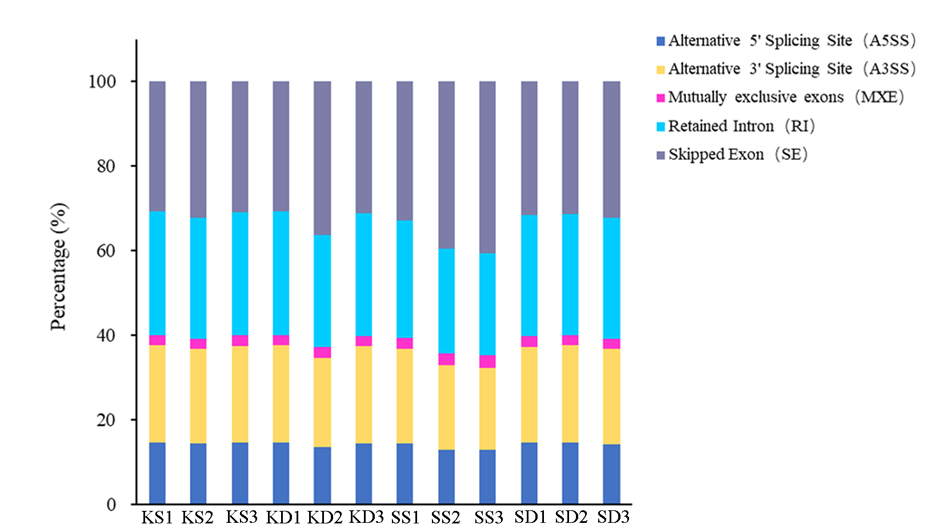

Supplement: Supplementary file 6 — Supplementary Figure S4. [file 41598_2020_72893_MOESM6_ESM.tif]

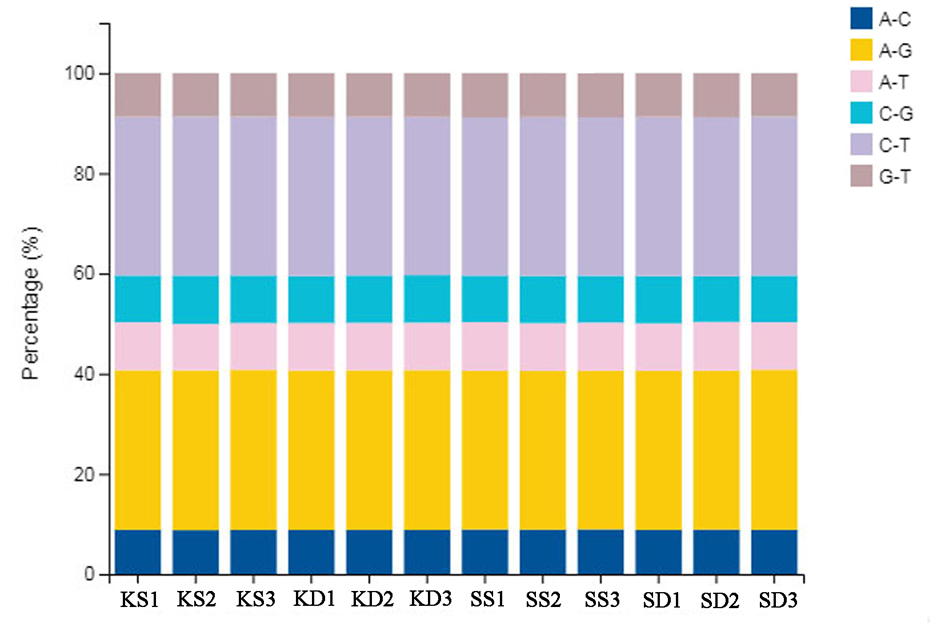

Supplement: Supplementary file 7 — Supplementary Figure S5. [file 41598_2020_72893_MOESM7_ESM.tif]

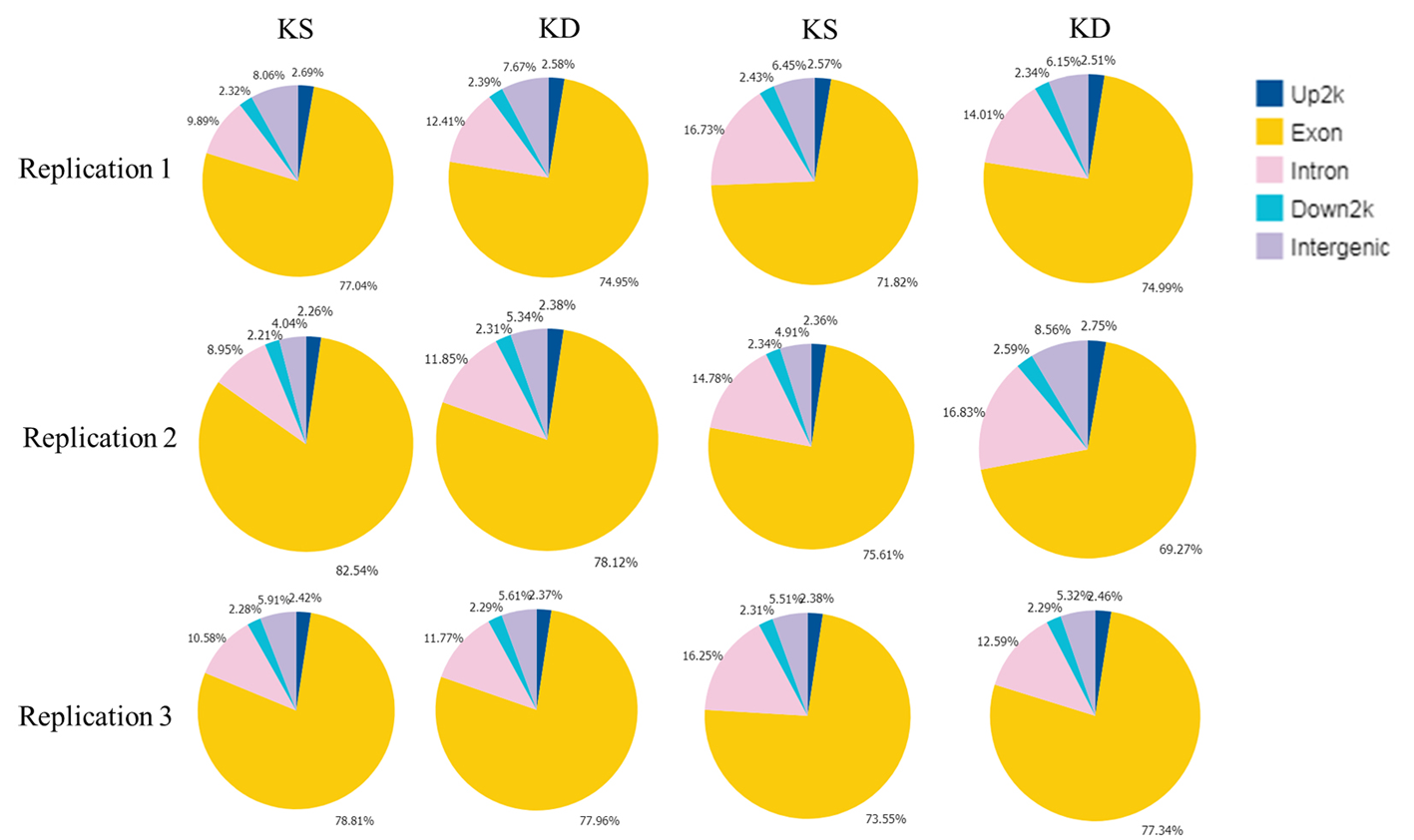

Supplement: Supplementary file 8 — Supplementary Figure S6. [file 41598_2020_72893_MOESM8_ESM.tif]

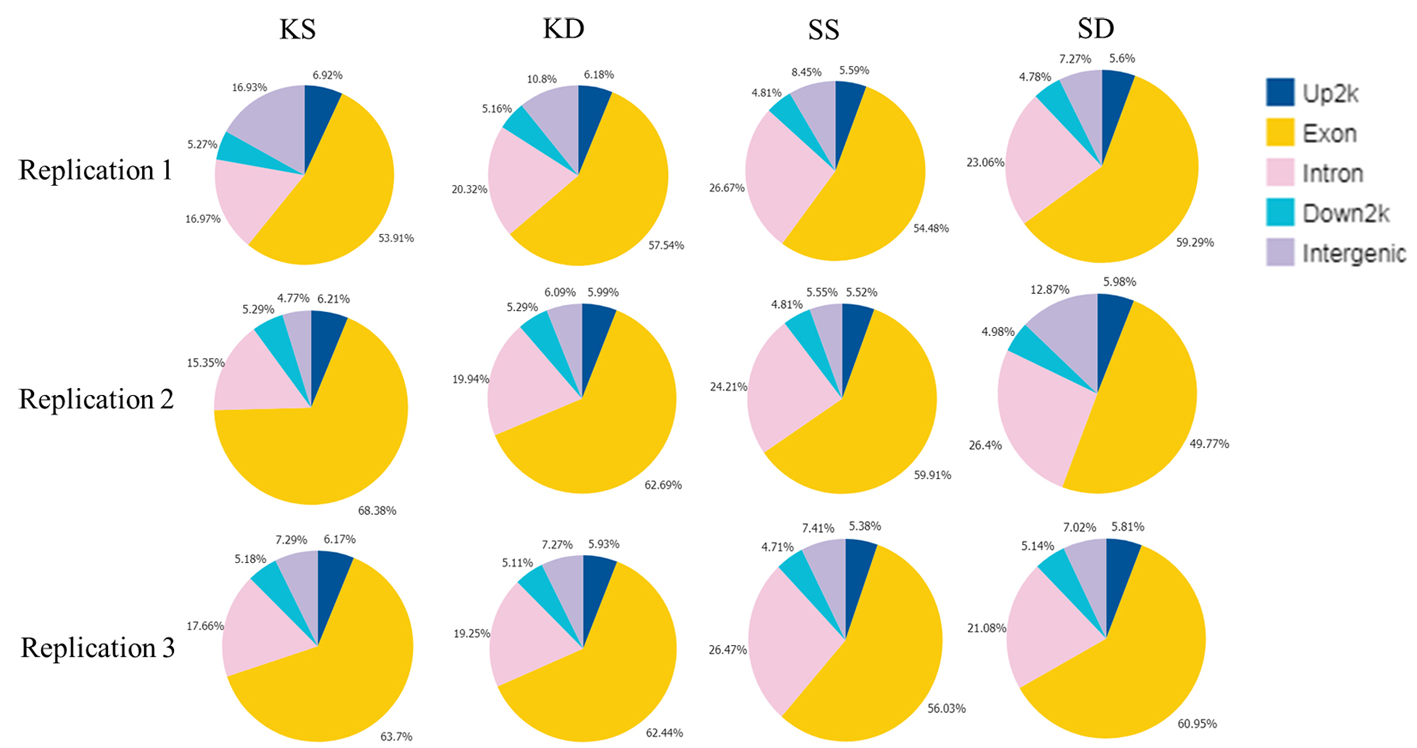

Supplement: Supplementary file 9 — Supplementary Figure S7. [file 41598_2020_72893_MOESM9_ESM.tif]
