# Supplementary material for: Transcriptome of peanut kernel and shell reveals the mechanism of calcium on peanut pod development
Source: Sci Rep. 2020 Sep 24;10:15723. doi: 10.1038/s41598-020-72893-9 (PMC7518428; doi:10.1038/s41598-020-72893-9)
Supplement: Supplementary file 10 — Supplementary Table S1. [file 41598_2020_72893_MOESM10_ESM.docx]

| **Gene Name** | **Primer** | **Primer Sequence (5’→3’)** |
| --- | --- | --- |
| Araip.MP018.1 | F | GTAGATGCGGGACACACGGTTC |
|  | R | AACCAATGGCGGGCTTTGAGG |
| Araip.37TY6.1 | F | AAAGGGTTGTCGCCTTGTTGT |
|  | R | AACCACCCTTTGGGAACTGC |
| Araip.AKR06.1 | F | TGGGGAAGGTACGCTGCTGAG |
|  | R | TGCGTCCTCGGCGGTGTC |
| Araip.C9B5T.1 | F | GCCAATGGAGCCATAGCCGAAG |
|  | R | ACGAGGTCTTGCCGCCAGAG |
| Araip.QB13B.1 | F | CCGCAGCCGAGGACAACAAC |
|  | R | TCACCGCCTCCGCCACTG |
| Araip.DW9H3.1 | F | GCGTCTGGAGGATACCAAAGCC |
|  | R | GCGTTTGAAGCTGTCGTTGCG |
| Araip.WXB7T.1 | F | ACGCACACGCCCCTGACTC |
|  | R | CGAACCGCCTTGACTCCACAC |
| Araip.JJU0M.1 | F | TCACAGCGGCAACAAGGTCAC |
|  | R | CGGCGACGATGAGGCTTCTTG |
| Araip.XKX17.1 | F | AGCTGGGTTCAGGTCCAAGGG |
|  | R | CCGTGTCAACCCTTCCATTCCC |
| Araip.1E1WQ.1 | F | GAAGACACACGTGCACCCAA |
|  | R | GGTTCTTCTCCAACACTGGCA |
| Araip.UK2VD.1 | F | GCTTCCGTGGACGACATAGCG |
|  | R | GGCCTGCCCACCCGTAGAG |
| Araip.AI6C6.1 | F | ACGCCGTTATGTGCTTCCGAAC |
|  | R | TGCGCTTTCACTGGCTCTTGC |
| Araip.BA4XW.1 | F | ATGGTTGGCGCTTCCCTTTGG |
|  | R | GGTCCTGCCGCTTGATCCAATG |
| Araip.313P6.1 | F | TGACCCAAGCACCAACCAAACC |
|  | R | ATGATCCATCACCGCTCACTGC |

**Table S1** Gene-specific primers used in quantitative real-time PCR.
